# The Unseen Burden: Uncovering Shame and Its Determinants in Parkinson's Disease

**DOI:** 10.1002/mdc3.70128

**Published:** 2025-05-19

**Authors:** Sabina Catalano Chiuvé, Damien Benis, Charline Rime, Maria João Forjaz, Paul Krack, Carmen Rodriguez‐Blazquez, Vanessa Fleury

**Affiliations:** ^1^ Department of Neurology Geneva University Hospital Geneva Switzerland; ^2^ Neuroscience of Emotion and Affective Lab, University of Geneva Geneva Switzerland; ^3^ Faculty of Psychology, University of Geneva Geneva Switzerland; ^4^ Faculty of Medicine, University of Geneva Geneva Switzerland; ^5^ National Centre of Epidemiology, Carlos III Institute of Health, REDISSEC Madrid Spain; ^6^ Center for Networked Biomedical Research in Neurodegenerative Diseases (CIBERNED), Carlos III Institute of Health Madrid Spain; ^7^ Department of Neurology Inselspital, University Hospital Bern, University of Bern Bern Switzerland

**Keywords:** embarrassment, health‐related quality of life, Parkinson's disease, shame, stigma

## Abstract

**Background:**

Shame is frequent in Parkinson's disease (PD) and often overlooked.

**Objective:**

The aim was to assess factors associated with PD‐related shame.

**Methods:**

PD‐related shame was measured using the Shame and Embarrassment in PD (SPARK) scale in patients without cognitive impairment. Correlation between *personal determinants* (demographics, psychological traits [shame/guilt propensity, trait anxiety]), *PD‐related determinants* (PD characteristics; motor, cognitive, and neuropsychiatric symptoms; medication; and health‐related quality of life [QoL]), and SPARK was analyzed using multiple correlation analysis and generalized linear mixed models. To describe the cohort's response to shame, data‐driven clustering based on SPARK was conducted, and clusters' associations with the determinants were analyzed.

**Results:**

Forty‐seven PD patients were included. PD‐related shame correlated with *psychological traits* (trait anxiety, shame, and guilt propensity), *clinical symptoms* (dyskinesia, state anxiety, depression, and apathy), and *QoL*. These determinants explained 79.3% of the total variance in the subsequent linear model analysis, with QoL and anxiety as the strongest covariates of shame. Apathy positively covaried with SPARK self‐esteem subscale. Cluster analysis identified 3 patient groups. Highest‐intensity shame cluster demonstrated elevated scores on both motor and nonmotor symptom–related shame and was associated with higher levels in anxiety, depression, and apathy, and poorer QoL. The remaining clusters showed a dissociation, with motor symptom–related shame predominating in one and nonmotor symptom–related shame in the other.

**Conclusions:**

This study provides an in‐depth understanding of shame, highlighting its multifactorial nature. Due to its impact on QoL, shame should be addressed in clinical practice through pharmacological/nonpharmacological interventions, targeting both shame and its modifiable determinants. Identifying distinct shame profiles underscores the need for tailored interventions.

Shame represents a significant source of emotional distress in patients living with Parkinson's disease (PD), affecting approximately 25% of them and substantially impacting their quality of life (QoL).^1^, ^2^ However, it is often neglected in clinical practice, possibly due to time constraints, limited resources, or a tendency among physicians to focus primarily on managing physical symptoms of PD rather than integrating emotional issues.

Shame is a negative self‐conscious emotion centered on a painful sense of self and may be influenced by others’ perceptions. It involves a sense of worthlessness and a need to hide.^3^ The distinction between shame and embarrassment remains debated; however, there are arguments for considering both emotions as part of the same emotional spectrum, with embarrassment involving a lower level of intensity and physical reaction than shame.^4^, ^5^ In the context of PD, shame results from the patient's perception that PD symptoms result in rule‐breaking behaviors/transgression. Nijhof^6^ identified 3 conditions that induce shame: (1) the rule is related to an important social value/competence, (2) the visibility of the behavior/symptom, and (3) the assumption of being labeled as a deviant. To extend this model, it is important to add that shame is experienced when the transgression is believed to define one's identity.^7^ Therefore, patients may experience a disconnection between their self‐image, their ideal self‐image, and the image they believe to project, resulting in an identity conflict. Transgressions involve not only social norms and visible symptoms but also personal values and nonvisible symptoms (eg, cognitive deficit). Although shame and stigma are related, they are conceptually distinct. Stigma is a complex social phenomenon encompassing stereotypes, prejudice, discrimination, and exclusion.^8^ Efforts to address stigma typically focus on public awareness and education to challenge misconceptions.^1^, ^9^ Furthermore, self‐stigma is tied to shame as it implies internalization of social stereotypes.^10^ Whereas self‐stigma is linked to societal perceptions, shame is an emotional response that can exist independently of social stereotypes, resulting from a perceived discrepancy between a situation and one's personal values or expectations. As an emotional response, shame should be modifiable through appropriate therapy. Identifying its determinants is crucial not only for providing an in‐depth understanding of this emotion but also for recognizing modifiable factors among them that could also serve as therapeutic targets. Our study focuses on shame because patients report that it has a profound impact on their QoL,^1^ highlighting the need for progress in its treatment.

This cohort study aimed to quantify and provide insights into the factors contributing to PD‐related shame, measured using the Shame and Embarrassment in PD (SPARK) scale, resulting from a co‐creation process between PD patients, clinicians, and scientists.^2^ Whereas social aspects are typically considered in stigma research,^9^, ^10^, ^11^, ^12^ this study specifically emphasized personal and PD‐related determinants to understand the internal factors contributing to shame in PD, which may be modifiable and relevant for therapeutic interventions. In this context, potential personal and PD‐related determinants of shame were identified based on studies focusing on shame in PD,^1^, ^2^, ^6^ drawing particularly on patients' and clinicians’ experiences.

## Patients and Methods

### Participants

Patients were recruited from the Movement Disorders Unit of the Geneva University Hospital and had a diagnosis of PD based on the United Kingdom Parkinson's Disease Society Brain Bank criteria.^13^ Exclusion criteria included neurocognitive impairment defined by a Montreal Cognitive Assessment (MoCA) score <26 of 30,^14^ structural brain disease other than PD, ongoing depression with suicidal ideation, clinically meaningful uncontrolled physical disease, and participation in a pharmacological study.

### Procedure

Patients were examined under their usual dopamine replacement therapy (DRT). To specifically study shame in PD using a quantitative approach, the level of shame was measured using the SPARK scale resulting from a co‐creation process between patients, clinicians, and scientists (Supplementary Material, sections S1 and S2).^2^ The SPARK scale has acceptable and reliable psychometric properties and measures the severity of shame and embarrassment related to PD, assessed through 33 items and a 4‐point Likert‐type response scale. Higher SPARK scores indicate higher levels of shame, with a maximum score of 99 points. SPARK subscales include shame resulting from PD symptoms (motor and nonmotor), shame resulting from increased physical dependence, shame resulting from body image deterioration, and consequences of PD on patient's self‐esteem and self‐stigmatization.

To the best of our knowledge, no study has specifically investigated shame determinants in PD. A self‐stigma study^10^ used a mixed methods design (qualitative and quantitative literature review; expert consultation) to identify determinants based on the ICF framework (International Classification of Functioning, Disability and Health [ICF] 2002), highlighting personal factors (eg, age, anxiety, apathy) and social aspects. In the absence of a specific scale assessing self‐stigma in PD, the 4‐item stigma subscale of the 39‐item Parkinson's Disease Questionnaire (PDQ‐39) was used,^11^, ^12^ and the predictors of PD self‐stigma identified were age^11^ and depression.^11^, ^12^

Since the few studies emphasizing shame in PD^1^, ^2^, ^6^ highlight its significant impact on QoL and its association with emotional distress, depression, anxiety, apathy, and personality traits, we explored these potential determinants. Shame, as a complex emotion, relies on cognitive capacities like self‐awareness and self‐representation,^15^ making it essential to also examine its link with global cognitive efficiency. As mentioned earlier, shame is an emotional response that can exist independently of social stereotypes, resulting from a conflict between a situation and personal values. Therefore, the focus was on personal and PD‐related determinants rather than social aspects. However, sociodemographic factors, such as education level, were also considered.

We defined the following potential determinants of shame:

1. *Personal determinants*, comprising sociodemographic data (gender, age, laterality, education level) and psychological traits (propensity to feel ashamed or guilty assessed using the Personal Feelings Questionnaire [PFQ‐2]^16^ and trait‐anxiety subscore using the State–Trait Anxiety Inventory [STAI]^17^). These scales have not been specifically studied in a population of PD patients. However, strong correlations have been demonstrated between SPARK and both the STAI and PFQ‐2.^2^ Also Rasch analysis suggests adjustments to improve STAI psychometric properties.^18^
2. *PD‐related determinants*, comprising (a) PD characteristics (duration, body side predominantly affected, Hoehn and Yahr [H&Y] stage^19^); (b) PD clinical symptoms: (i) motor symptoms: severity of motor PD triad using the Movement Disorder Society‐Unified Parkinson's Disease Rating Scale III (MDS‐UPDRS)^20^ and severity of dyskinesia using the Marconi Dyskinesia Rating Scale (MDRS)^21^; (ii) nonmotor symptoms: global cognition using MoCA, a good discriminative tool for screening mild neurocognitive impairment in PD,^14^, ^22^ neuropsychiatric symptoms (apathy assessed using the Starkstein Apathy Scale [SAS],^23^, ^24^, ^25^ depression using the Beck Depression Inventory [BDI], both valid and reliable in PD patients,^26^, ^27^ anxiety using STAI state subscore^17^); (c) medication (expressed in levodopa‐equivalent daily dose [LEDD]^28^); and (d) health‐related QoL using the 8‐item PD Questionnaire (PDQ‐8).^29^

### Statistical Analysis

Based on the administered questionnaires selected to explore the aforementioned determinants, exploratory statistical analyses were conducted to examine their covariations with shame while minimizing type I errors using controlled analyses. For a more detailed description of the method, see Supplementary Material, section S3. The *P*‐values from models and contrasts were corrected using the false discovery rate (FDR) method.^30^

### Impact of Personal Determinants and PD‐Related Determinants on Total‐SPARK Score and SPARK Subscale Scores

To test the correlation of each personal and PD‐related determinant score with the total‐SPARK score, multiple Pearson's correlation analysis for continuous variables as well as analysis of variance (ANOVA) for categorical variables was performed on *JASP* (version 0.19.3). Variables were square root normalized to ensure the validity of the normality assumption (Shapiro–Wilk test for multivariate normality = 0.968, *P* = 0.371), and *P*‐values were FDR corrected (19 tests^30^). To explore whether a score covaried with shame in relation to its relationship to other personal and PD‐related determinant scores that significantly correlated with SPARK total scores, these determinant scores were entered as covariates into a single linear model, with SPARK total scores as dependent variables.

To verify whether the effect of a score presented a differential association between the SPARK subscales, scores from each SPARK subscale (PD symptoms: motor and nonmotor, physical dependance, body image, self‐esteem, stigmatization) were normalized as a proportion of the maximal score of the SPARK scale and entered into a generalized linear mixed model (Poisson distribution, *JASP* software).^31^ The 2‐way interaction between the trait value (covariate) and subscales title (factor) was computed. Trend contrast analysis was then performed to test the significance of the covariance of the score considered using each SPARK subscale. Model *P*‐values were corrected using FDR (19 tests^30^).

### Data‐Driven Clustering on SPARK Subscales and Statistical Characterization of Clusters

To further characterize the structure of our cohort response to shame, we performed a data‐driven hierarchical clustering on the SPARK subscales to sort our PD population according to the type of shame they experienced. First, data from all SPARK subscales were entered for performing principal component analysis with varimax rotation using the psych R package,^32^ and the number of components was determined using the elbow method. Factorial scores from the obtained components (Supplementary Material, section S3) were entered into a hierarchical clustering algorithm (psych R package) using Euclidean distance and Ward linkage (highest silhouette coefficient = 0.92), and the NClust R package^33^ for cluster number estimation. Differences between clusters in terms of total‐SPARK scores and SPARK subscale ratings were, respectively, tested using ANOVA and generalized linear models (GLM) (with patient as random factor in the second analysis), followed by contrast analysis (*JASP* software and glmmTMB R package). We then checked whether each cluster was characterized by a differential pattern of personal and/or PD‐related determinants, using a linear model with the trait of interest as a dependent variable and the cluster identity as the main effect, followed by contrast analysis. Finally, for continuous variables, we verified whether the covariance between shame and personal and/or PD‐related determinants differed between the different patient clusters. Data were entered into a linear model using the SPARK total score as a dependent variable, testing the 2‐way interaction between clusters and the determinant of interest. Contrast analysis was then performed, and *P*‐values from the models and contrasts were corrected via FDR.

## Results

### Patient Characteristics

A total of 47 PD patients were recruited. Demographic and clinical characteristics are presented in Table 1. PD duration was on average 9.8 years (standard deviation = 4.2 years), and 96% of patients were in H&Y stage 2 or above.

**TABLE 1:** Demographic and clinical characteristics of patients

	PD patients
Demographics	
Number of participants (men)	47 (22)
Mean age in years (SD)	65.1 (8.3)
Education level (N in I, II, ≥III^a^)	7, 16, 24
Mean PD duration in years (SD)	9.8 (4.2)
Body side predominantly affected by PD (N left/right)	29, 18
Mean Hoehn and Yahr stage (/4)	2.2 (<2 = 4%, 2 = 66%, >2 = 30%)
Clinical symptoms	
MDS‐UPDRS motor score (/132)	21.2 (10.8)
Dyskinesia Rating Scale (/28)	3.0 (3.5)
Cognitive score MoCA (/30)	28.3 (1,3)
PD‐related shame SPARK total score (/99)	26.1 (18.8)
Personal Feelings Questionnaire (PFQ‐2) total score (/64)	12.6 (10.0)
Propensity to feel ashamed subscale (/40)	7.1 (6.0)
Propensity to feel guilty subscale (/24)	5.5 (4.7)
STAI	
STAI state (/80)	28.6 (9.3)
STAI trait (/80)	35.9 (8.9)
BDI‐II (/63)	10.7 (5.9)
SAS (/42)	11.4 (5.2)
Health‐related quality of life (PDQ‐8) (/8)	10.1 (5.6)
Medication	
Levodopa‐equivalent daily dose (mg/day)	954.6 (472.7)

*Notes*: Clinical symptoms were assessed during chronic *on*‐drug state. Values are expressed as mean (SD) for continuous variables and frequency (percentage, %) for categorical variables. A SAS ≥14 of 42, an anxiety STAI score ≥50 of 80 for state and ≥48 of 80 for trait, and a BDI score ≥21 of 63 were pathological cutoff values.

^a^ Education level: level I is defined as subjects who received a primary education, level II a lower secondary education, and level ≥3 at least an upper secondary education.

Abbreviations: PD, Parkinson's disease; SD, standard deviation; N, number; MDS‐UPDRS, Movement Disorder Society‐Unified Parkinson's Disease Rating Scale; MoCA, Montreal Cognitive Assessment; SPARK, Shame and Embarrassment in PD; STAI, State–Trait Anxiety Inventory; BDI, Beck Depression Inventory; SAS, Starkstein Apathy Score; PDQ‐8, 8‐item Parkinson's Disease Questionnaire.

### PD‐Related Shame Distribution among Patients

**FIG. 1:**
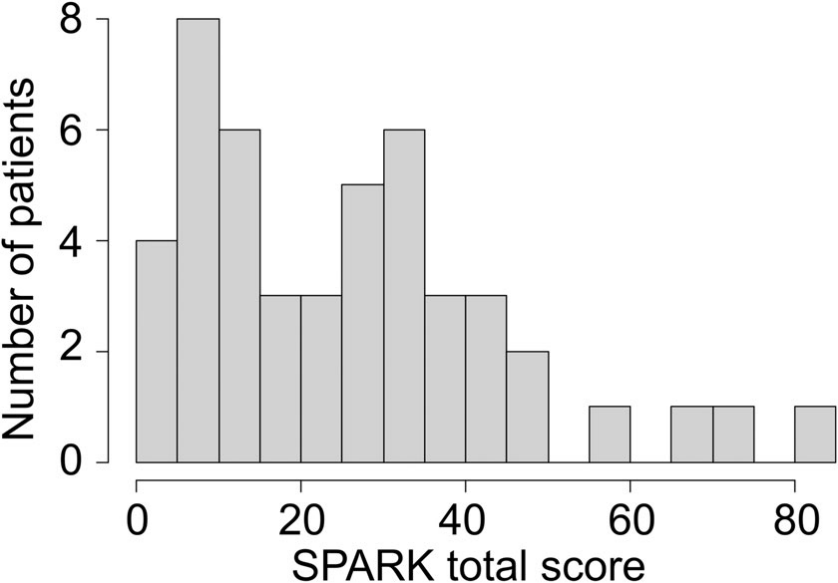
Patient distribution in relation to the intensity of total‐SPARK (Shame and Embarrassment in Parkinson's Disease) score.

The patient's distribution in relation to the intensity of total‐SPARK score is shown in Figure 1. Forty‐five percent of patients presented with a low shame intensity, 34% with a moderate level of shame, and 21% with severe shame.

### Impact of Personal Determinants and PD‐Related Determinants on Total‐SPARK Score

Multiple correlation analysis between PD‐related shame intensity and the personal and PD‐related determinants is presented in Table 2. A complete multiple correlation matrix is presented in Supplementary Material, section S4. Propensity to feel ashamed, guilty, or anxious positively correlated with PD‐related shame intensity, whereas the other personal determinants (age, gender, education level) did not show any statistically significant associations. In terms of PD‐related determinants, PD duration, H&Y stage, and body side predominantly affected by PD were not associated with PD‐related shame intensity. Regarding clinical PD symptoms, dyskinesia, anxiety, depression, and apathy positively correlated with PD‐related shame, whereas MDS‐UPDRS motor score and MoCA did not. There was no association between shame and LEDD. Highest correlation coefficient for PD‐related shame intensity was obtained for poor QoL. This effect remained significant even after accounting for the total variance in other clinical scores that significantly covaried with the SPARK total score using a linear model (multicollinearity and error independence assumptions were verified, Variance Inflation Factor (VIF) <5, tolerance >0.2, and Durbin–Watson score = 1.558), along with trait‐anxiety score (Table 3). Note that the determinants included in the model accounted for 79% of the total variance, implying a strong relevance of the determinants selected.

**TABLE 2:** Correlation between the intensity of PD‐related shame (SPARK total score) and personal and PD‐related determinants

Multiple correlation analysis	n	Pearson's r	*P*	*P* FDR corrected	Effect size (Fisher's z)	SE effect size	Significance
Personal determinants							
Demographic data							
Age	47	−0.239	0.106	0.159	−0.244	0.151	ns
Psychological traits							
Propensity to feel guilty (PFQ‐2)	35	0.509	0.002	0.004	0.562	0.177	**
Propensity to feel ashamed (PFQ‐2)	35	0.724	0.001	0.002	0.917	0.177	**
Trait‐anxiety (STAI‐trait)	35	0.758	0.001	0.002	0.991	0.177	**
PD‐related determinants							
PD characteristics							
Disease duration	47	0.064	0.669	0.723	0.064	0.151	ns
Hoehn and Yahr stage (/4)	47	0.005	0.973	0.973	0.005	0.151	ns
Clinical symptoms							
Motor symptoms							
Severity (MDS‐UPDRS III)	47	0.048	0.751	0.793	0.048	0.151	ns
Dyskinesia (Marconi)	47	0.446	0.002	0.004	0.479	0.151	**
Global cognition (MoCA)	47	−0.089	0.551	0.733	−0.089	0.151	ns
Neuropsychiatric symptoms							
State‐anxiety (STAI‐state)	47	0.512	0.001	0.002	0.566	0.151	**
Depression (BDI‐II)	35	0.723	0.001	0.002	0.914	0.177	**
Apathy (SAS)	47	0.457	0.001	0.002	0.493	0.151	**
Medication							
Levodopa‐equivalent daily dose (mg/d)	47	0.182	0.222	0.324	0.184	0.151	ns
Quality of life							
Health‐related quality of life (PDQ‐8)	47	0.792	0.001	0.002	1.075	0.151	**

ANOVA (categorical variables)	n	Mean square	*F*	*P*	*P* FDR Corrected	*η*²	Significance
Personal determinants							
Demographic data							
Gender	47	1.073	0.314	0.579	0.733	0.007	ns
Education level	47	1.318	0.385	0.683	0.763	0.018	ns
PD‐related determinants							
PD characteristics							
Body side predominantly affected	47	9.902	2.895	0.097	0.168	0.066	ns

*Notes*: Clinical symptoms were assessed during chronic ON‐drug state. ns = non‐significant. p FDR corrected values ≤ 0.05 are summarized with one asterisk (*), and p FDR corrected values ≤ 0.01 with two asterisks (**).

Abbreviations: PD, Parkinson's disease; SPARK, Shame and Embarrassment in PD; FDR, false discovery rate; ns, not significant; PFQ‐2, Personal Feelings Questionnaire; STAI, State–Trait Anxiety Inventory; MDS‐UPDRS, Movement Disorder Society‐Unified Parkinson's Disease Rating Scale; MoCA, Montreal Cognitive Assessment; BDI, Beck Depression Inventory; SAS, Starkstein Apathy Scale; PDQ‐8, 8‐item Parkinson's Disease Questionnaire; ANOVA, analysis of variance; SE, Standard Error.

**TABLE 3:** Linear model analysis for covarying and personal and PD‐related determinants with the intensity of PD‐related shame (SPARK total score)

Model	*R*	*R* ^2^	Adjusted *R* ^2^	RMSE
	0.917	0.842	0.793	8.434

Coefficients				
	Standard error	β	*t*	*P*
STAI trait	3.661	0.366	2.571	0.016
PDQ‐8	3.156	0.383	2.276	0.031
BDI	3.281	−0.282	−1.731	0.095
PFQ guilt	1.952	0	0.004	0.996
STAI state	3.266	0.185	1.312	0.201
MDRS	1.652	0.063	0.631	0.533
PFQ shame	2.137	0.242	1.722	0.097
SAS	3.176	0.211	1.693	0.102

Abbreviations: PD, Parkinson's disease; SPARK, Shame and Embarrassment in Parkinson's Disease scale, assessing PD‐related shame; RMSE, root mean square error; STAI TRAIT, trait‐anxiety subscale of State–Trait Anxiety Inventory; PDQ‐8, 8‐item Parkinson's Disease Questionnaire, assessing quality of life; BDI, Beck Depression Inventory, assessing depression; PFQ guilt, guilt subscale of Personal Feelings Questionnaire (PFQ‐2); MDRS, Marconi Dyskinesia Rating Scale, assessing dyskinesia; SAS, Starkstein Apathy Scale, assessing apathy.

### Impact of Personal and PD‐Related Determinants on SPARK Subscores

A significant interaction between SPARK subscales and determinants was observed only for the apathy score (SAS) using GLM with Poisson distribution. Contrast analysis revealed that apathy score positively covaried with the SPARK self‐esteem subscore (χ^2^ = 24.639, *P* = 0.015 [FDR corrected]). No other interactions were found.

### PD Patient Clusters

After our main experimental question was addressed, we took an additional step by exploring potential patient profiles using clustering analysis on SPARK subscales. Hierarchical clustering of dimension‐reduced SPARK subscores (Supplementary Material, section S3) yielded 3 clusters differing in terms of SPARK total score (ANOVA, *F*(2) = 56.474, *P* < 0.001, *η* ^2^ = 0.751) (Supplementary Material, section S5): the *highest total‐SPARK score cluster* had equally high shame secondary to motor and nonmotor symptoms, the *intermediate total‐SPARK score cluster* had shame mainly linked to motor symptoms, and the *lowest total‐SPARK score cluster* had shame mainly linked to nonmotor symptoms (Supplementary Material, section S6).

Regarding the influence of personal and/or PD‐related determinants on patient's clusters, GLM analysis (Supplementary Material, section S7) revealed inter‐patient cluster difference for shame propensity (PFQ‐2), dyskinesia severity (MDRS), levels of anxiety (STAI), depression (BDI), apathy (SAS), and QoL (PDQ‐8), mainly following the SPARK total profile, with highest scores observed in the highest total‐SPARK score cluster. The difference in MDRS and shame PFQ‐2 is significant only for the lowest total‐SPARK score cluster, which is significantly lower on both measures compared to the other 2 clusters (Supplementary Material, section S8).

## Discussion

Despite its significant impact on QoL in PD, shame remains underexplored.^1^ This study investigated personal and PD‐related determinants involved in PD‐associated shame and underscores the fact that PD‐related shame is a multifactorial entity. The main personal determinants were related to personality traits, such as the propensity to feel ashamed, guilty, or anxious. Regarding PD characteristics, dyskinesia, anxiety, depression, and apathy positively correlated with PD‐related shame, whereas PD duration, H&Y stage, body side predominantly affected by PD, MDS‐UPDRS motor score, MoCA score, and LEDD did not. PD‐related shame was strongly associated with poor QoL and trait anxiety. The clustering analysis identified 3 groups of patients, with some patients more affected by shame in relation to their motor symptoms than their nonmotor symptoms, and vice versa. Patients with higher levels of shame were affected by both motor and nonmotor symptom–related shame and presented more apathy, depression, and anxiety, and poorer QoL. These determinants were consistently identified in both the correlation and clustering analyses.

Because shame might hinder the process of accepting the disease, it represents an obstacle to proper adjustment to PD, which can lead to decreased life satisfaction, drug intolerance, and poorer QoL.^34^, ^35^, ^36^ If the patient reports PD‐related shame with a distressing functional or psychological impact, shame should be considered a disabling symptom of PD and integrated into the management plan. It is worth noting that shame is a moral emotion and can be perfectly functional, serving as a social regulator.^37^ However, when the impact of shame becomes overwhelming or maladaptive, it is essential to address it and offer not only pharmacological treatment but also nonpharmacological options, such as psychoeducation and psychotherapy.^38^, ^39^

The predominant personal determinants were the proneness to feel ashamed or guilty, and trait anxiety. They represent possible modifiable factors,^40^ as they can be attenuated with psychosocial intervention such as group or individual therapy and psychoeducation. Cognitive behavioral therapy (CBT) could be proposed to help the individual to identify and change negative or distorted thought patterns that contribute to shame and feelings of identity loss.^39^, ^41^, ^42^ Such CBT could also focus on developing an attitude of kindness and understanding toward oneself, even in the face of difficulties and failures. This involves learning to accept oneself with compassion rather than harsh self‐criticism.

Depression, anxiety, and apathy were interrelated symptoms that positively covaried with shame and poorer QoL. There are also modifiable factors that can be addressed using a multidisciplinary and coordinated pharmacological and nonpharmacological treatment approach. Notably, psilocybin‐assisted psychotherapy helps shift from emotional avoidance of difficult emotions to acceptance,^43^ which could be promising in improving PD patients’ well‐being.^44^

The severity of dyskinesia positively covaried with the level of shame in non‐anosognosic PD patients with dyskinesias. Dyskinesias should therefore not be trivialized. Whenever they are present, levodopa therapy should be adjusted accordingly.

Apathy was also found to correlate with shame, particularly in relation to loss of self‐esteem. In clinical practice, apathetic patients often express a sense of shame and low self‐esteem. They feel capable of doing various things but are concretely incapable of carrying out activities due to apathy.^45^

Clustering analysis demonstrated that PD‐related shame is a multifaceted entity. It uncovered 3 groups of patients: one where shame was predominantly secondary to motor symptoms, one where shame secondary to nonmotor symptoms predominated over shame secondary to motor symptoms, and one where shame secondary to motor and nonmotor symptoms was equally high. This dissociation offers the potential to disentangle the putative cerebral networks underlying motor and nonmotor symptom–related shame (see ClinicalTrials.gov NCT06225869).

Shame and embarrassment in PD are rarely explored, not only in routine clinical practice but also in PD research. A possible explanation is that there is some ambiguity in the definition and the terms employed. Several publications focus on self‐stigma^10^, ^11^, ^12^, ^46^ rather than shame. The concept of self‐stigma is related but different from that of shame. Self‐stigma results from the misattributions of PD symptoms that are erroneously associated with a negative stereotype. PD patients internalize this socially devalued status.^10^ Shame is the emotional core of the experience of stigma.^47^ Shame differs from the concept of self‐stigma that originates in the social context, whereas shame originates in the individual themselves. Shame not only involves a social dimension but also a personal, intimate dimension, and a perception of violating personal norms. Whereas self‐stigma pertains to the adoption of negative beliefs, stereotypes, and prejudices associated with the illness, shame entails a negative self‐evaluation due to perceived inadequacies, mistakes, or actions contrary to personal or social values. Both shame and self‐stigma are frequent^2^, ^12^ and associated with poorer QoL and depression.^11^, ^12^, ^48^ A mixed methods scoping review exploring determinants of self‐stigma in PD identified 87 determinants of self‐stigma.^10^ Quantitative studies and expert consultations identified personal determinants such as age, anxiety, or apathy, as being associated with self‐stigma, whereas qualitative studies highlighted social determinants. PD characteristics such as cognitive impairment, tremor, and gait disturbances were the main symptoms associated with self‐stigma across methods.^10^ Younger age in men and depression have been identified as the primary predictors of self‐perceived stigma in PD.^11^ Age and depression seem to be the consistent determinants of self‐stigma. Depression thus appears to be a factor associated with both shame and self‐stigma. Our findings further indicate that, in PD‐related shame, personality traits, anxiety, and apathy contribute independently of disease severity, duration, and antiparkinsonian treatments. The determinants identified in this study are thus psychological, except for dyskinesias, which are motor symptoms. However, when patients are aware of them, dyskinesias can reinforce a feeling of loss of control over their own body and may not align with the image they wish to project. The influence of social exposure should be further explored in future research.

This study has certain limitations. First, the sample size is relatively small, and further confirmatory studies should be conducted to validate these results in larger samples. The sample size was initially based on patient availability. Although the sample size may be underpowered, the linear model analysis, which included the positive results from our multiple correlations, accounted for 79% of the total variance of the dependent variable (SPARK). This suggests that the positive results of the correlation addressed the study experimental question, that is, the determinants of shame in PD, quite extensively. However, future studies may be needed to test the stability and replicability of the current study. Second, we focus primarily on the internal determinants of shame, whereas the external and environmental factors were considered in less detail. Anxiety, depression, and apathy were considered secondary to PD, but they could also result from other causes. The study relies on a questionnaire based on patients' memory, rather than capturing their immediate emotional experience or perspective taking. This reliance on self‐reported data introduces the potential for recall bias, which may have influenced the findings. Future research could benefit from studying shame and embarrassment in PD using a tailored shame induction task (see ClinicalTrials.gov NCT05119075). Moreover, motor evaluation was based on UPDRS III, using objective criteria, but it did not assess the patient's subjective experience as UPDRS II does. However, self‐reported measures could be influenced by shame, leading to spurious correlation. A controlled study is needed to explore the correlation between shame and the discrepancy between objective and subjective symptom severity. Additionally, patients were examined under their usual DRT. It would be valuable to investigate how the ON‐ and OFF‐DRT states might differentially impact the experiences of shame and embarrassment (see NCT05119075). Moreover, the integration of magnetic resonance imaging would help to better understand the neuroanatomical underpinnings of shame in PD (see NCT06225869). Finally, our patient group was homogeneous in terms of disease stage and disease duration, and no patient had significant cognitive impairments. It would be interesting to study the level of shame based on the disease duration and to compare the level of shame in the different stages of the disease.

In conclusion, this study provided an in‐depth understanding of shame, particularly its multifactorial nature. Due to its severe impact on patients' QoL, shame should be properly addressed in clinical practice. This study offers valuable insights into its management using pharmacological and/or nonpharmacological interventions, enabling targeted approaches that address not only shame as a modifiable emotional response but also its modifiable determinants, including shame or guilt propensity, anxiety, depression, and apathy. Identifying distinct shame profiles underscores the need for tailored interventions.

## Author Roles

1. Research project: A. Conception, B. Organization, C. Execution;
2. Statistical analysis: A. Design, B. Execution, C. Review and critique;
3. Manuscript preparation: A. Writing of the first draft, B. Review and critique.

S.C.C.: 1A, 1B, 1C, 2C, 3A

D.B.: 1C, 2A, 2B

C.R.: 1A, 2C, 3B

M.J.F.: 1A, 2C, 3B

P.K.: 1A, 2C, 3B

C.R.‐B.: 1A, 2C, 3B

V.F.: 1A, 1B, 1C, 2C, 3B

## Disclosures

**Ethical Compliance Statement**: This study was approved by the Geneva Ethics Committee (CCER: numbers 2019‐01026 and 2021‐01608). All participants provided written informed consent, and all methods were performed in accordance with the Declaration of Helsinki. All authors confirm that they have read the journal's position on issues involved in ethical publication and affirm that this work is consistent with those guidelines.

**Funding Sources and Conflicts of Interest:** This study was partially supported by the Foundation Bertarelli Catalyst Fund. The authors declare that there are no conflicts of interest relevant to this work.

**Financial Disclosures for the Previous 12 Months:** The authors declare that there are no additional disclosures to report.

## Supporting information


**Data S1.** Supporting information.
