# Supplementary material for: The Unseen Burden: Uncovering Shame and Its Determinants in Parkinson's Disease
Source: Mov Disord Clin Pract. 2025 May 19;12(10):1551–9. doi: 10.1002/mdc3.70128 (PMC12528978; doi:10.1002/mdc3.70128)
Supplement: Supplementary file 1 — Data S1. Supporting information. [file MDC3-12-1551-s001.docx]

**Supplementary material**

1. **The SPARK scale**

Embarrassment and shame can be experienced with Parkinson’s disease. These emotions can be the source of emotional distress and impact on quality of life.

For each statement, please tick the response that best describes the way you have been feeling recently.

Please take into account the beginning of the sentence marked in bold for each of the following questions.

| **I feel embarrassed or ashamed due to Parkinson’s disease …** | | | | |
| --- | --- | --- | --- | --- |
| **… because of my visible symptoms such as:** |  |  |  |  |
| 1. Tremor | No | A little | Moderately | A lot |
| 1. Slowness, small writing, lack of dexterity, stiffness | No | A little | Moderately | A lot |
| 1. Dry mouth or excess saliva | No | A little | Moderately | A lot |
| 1. Walking, balance or postural difficulties | No | A little | Moderately | A lot |
| 1. Jerking or involuntary movements | No | A little | Moderately | A lot |
| 1. … because others have an image of me that doesn’t correspond to who I really am. | No | A little | Moderately | A lot |
| 1. … because I need to ask for help from my family or people around me. | No | A little | Moderately | A lot |
| 1. … because my speech difficulties (articulation, voice, rhythm, understandability) affect my ability to communicate with others. | No | A little | Moderately | A lot |
| 1. … because this disease is perceived to be a disease of older people. | No | A little | Moderately | A lot |
| 1. … because I am no longer able to do what I once did in the past. | No | A little | Moderately | A lot |
| 1. … because my lack of facial expression is misinterpreted as being unfriendly, odd or ill-tempered. | No | A little | Moderately | A lot |

| **I feel embarrassed or ashamed due to Parkinson’s disease …** | | | | |
| --- | --- | --- | --- | --- |
| **… because of my non motor symptoms such as:** |  |  |  |  |
| 1. Modification of emotions (over sensitivity, blunting) | No | A little | Moderately | A lot |
| 1. Problems with memory or concentration difficulties, or slow thinking | No | A little | Moderately | A lot |
| 1. Hallucinations | No | A little | Moderately | A lot |
| 1. Behavioral changes (e.g., gambling addiction, hypersexuality, compulsive buying, boulimia) | No | A little | Moderately | A lot |
| 1. Reduced motivation | No | A little | Moderately | A lot |
| 1. … because people think that I am more fragile than I really am. | No | A little | Moderately | A lot |
| 1. … because I am progressively losing my independence. | No | A little | Moderately | A lot |
| 1. … because my body no longer represents who I really am. | No | A little | Moderately | A lot |
| 1. … because of my urinary problems (urgent need to go to the toilet), stomach problems (e.g., constipation) or my sexual difficulties. | No | A little | Moderately | A lot |
| 1. … because I am losing control of my body. | No | A little | Moderately | A lot |
| **Due to Parkinson’s disease, …** |  |  |  |  |
| 1. … I feel useless. | No | A little | Moderately | A lot |
| 1. … some people seem uncomfortable around me. | No | A little | Moderately | A lot |
| 1. … people avoid looking at me. | No | A little | Moderately | A lot |
| 1. … I feel worthless. | No | A little | Moderately | A lot |
| 1. … I feel incompetent. | No | A little | Moderately | A lot |
| 1. … I avoid going out, talking in public or social encounters. | No | A little | Moderately | A lot |
| 1. … I feel left out of things. | No | A little | Moderately | A lot |
| 1. … I am not being taken seriously. | No | A little | Moderately | A lot |
| 1. … I feel that I have to hide my disease. | No | A little | Moderately | A lot |
| 1. … I feel worried by others’ reaction to me. | No | A little | Moderately | A lot |
| 1. … I feel embarrassed. | No | A little | Moderately | A lot |
| 1. … I feel ashamed. | No | A little | Moderately | A lot |

1. **Instructions for administration and scoring of the SPARK scale**

The scoring system is as follows:

No = 0 point; A little = 1 point; Moderately = 2 points; A lot = 3 points.

**Total score:** /99

**Subscales:**

1. Embarrassment and shame arising from Parkinson’s disease symptoms:

Motor symptoms: Items 1, 2, 4, 5, 8, 11: /18

Non-motor symptoms: items 3, 12, 13, 14, 15, 16, 20: /21

Subscore: /39

1. Embarrassment and shame arising from the increasing physical dependence and need for help induced by Parkinson’s disease:

Items 7, 10, 18

Subscore: /9

1. Embarrassment and shame arising from a deteriorated body image:

Items 6, 9, 17, 19, 21

Subscore: /15

1. Consequences of Parkinson’s disease on patients’ self-esteem:

Items 22, 25, 26

Subscore: /9

1. Self-stigmatization

Items 23, 24, 27, 28, 29, 30, 31:

Subscore: /21

1. Types and intensity of self-evaluative negative emotion:

Item 32 for embarrassment: /3

Item 33 for shame: /3

Fleury V, Catalano Chiuvé S, Forjaz MJ, Di Marco M, Messe M, Debove I, et al. Embarrassment and Shame in People With Parkinson’s Disease: A New Tool for Self-Assessment. Frontiers in Neurology. 2020;11:779.

1. **Statistical analysis**

*Impact of personal determinants and PD-related determinants on total SPARK score and SPARK subscale scores*

Square-root total SPARK score was entered into 19 linear models using the lme4 R function to test the main effect of each *personal determinant and PD-related determinant* score on the SPARK score. The square-root normalization procedure allowed to get closer to a normal distribution. Akaike Information Criterion (AIC) analysis showed that the model with the square-root normalization and linear distribution yielded the lowest AIC compared to normalized and non-normalized Poisson and Tweedie models and non-normalized linear models. *p*-values obtained for these 19 models were corrected using False Discovery Rate (FDR) (Haynes W, 2013).

To test whether a score covaried independently from other scores, a supplementary analysis was undertaken. Scores were entered into a linear model with the other scores presenting independently a significant effect on total SPARK score in the previous analysis as covariates. If significance persisted after adding the covariates, it indicated that a part of the variance for the effect of the score was independent from the other scores tested in the previous analysis. Finally, to check whether the effect of a score presented a differential covariation between the SPARK subscales, scores from each SPARK subscales (PD symptoms: motor and non-motor, Physical dependance, Body image, Self-esteem, Stigmatization), were normalized as proportion of the maximal score of the SPARK scale and entered into a Generalized Linear Mixed model (GLMM). A Tweedie distribution was chosen to account for the 0 inflation and overdispersion of the distribution. The two-way interaction between the trait-value and scale was computed with “Patient” as random factor using the GLMM Template Model Builder package. Contrast analysis was then performed on trends using the emtrend function of the emmeans package to check: 1) The significance of the covariation of the score considered with each SPARK subscale and 2) Whether the trend of the covariation with the score differed between each subscale.

*Data-driven clustering on SPARK subscales*

To further characterize the structure of our population response to shame, we performed a data driven clustering on the SPARK subscales to sort our PD population according to the type of shame they experience. First, data from all SPARK subscales were entered into a principal component analysis (PCA) with varimax rotation using the psych R package. The number of components were determined using the elbow method. Factorial scores from the obtained components were entered into a hierarchical clustering algorithm. The best distance metric and linkage were determined using the silhouette coefficient and the optimal number of clusters were determined using the NbClust R packages using 30 indices for determining the optimal number of clusters (see Table below).

Components saturation after VARIMAX rotation

|  | **Component 1** | **Component 2** | **Component 3** |
| --- | --- | --- | --- |
| **SPARK subscales** |  |  |  |
| Motor PD Symptoms | **0.84** | 0.28 | 0.31 |
| Non-motor PD symptoms | 0.36 | 0.20 | **0.87** |
| Physical dependency | 0.36 | **0.88** | 0.25 |
| Body Image | **0.79** | 0.45 | 0.24 |
| Self-esteem | 0.20 | **0.61** | **0.66** |
| Stigmatization | **0.88** | 0.16 | 0.22 |

PCA on SPARK subscores yielded three components explaining 89% of the total variance in the sample. One component saturated on shame related to “motor PD symptoms” as well as “body image” and “stigmatization” (Component 1). A second component saturated on “physical dependency” as well as “self-esteem” (Component 2) and a third component saturated on shame linked with “non-motor PD symptoms” as well as “self-esteem” (Component 3). To simplify and summarize, Component 1 is named "motor", Component 2 "non-motor", and Component 3 “physical dependency and self-esteem” in the manuscript.

*Statistical characterization of clusters*

First, the total SPARK score was compared between the three-groups (low, medium and high shame), using a GLM with the cluster identity as factor and FDR corrected pairwise contrasts were performed. Then the normalized SPARK subscales were entered into a GLMM with a Tweedie distribution (see above) to test the interaction between cluster membership and SPARK subscale on SPARK total score with patient ID as random factor. Contrast analyses were then performed and corrected using FDR.

Secondly, we checked whether each cluster was characterized by a differential pattern of personal and PD-related determinants. Data was entered into a GLM with the determinant of interest as dependent variable and the cluster identity as main effect. The distribution of the GLM was determined by comparing the AIC of a poisson, gaussian and tweedie model wih or without square root normalization of the dependent variable. The model with the lowest AIC was chosen for further analysis. Contrast analysis was then performed and *p*-values from the models and contrasts were corrected via FDR.

Thirdly, we checked for continuous variables whether the covariation between shame and personal and PD-related determinants differed between the different population clusters. Data was entered into a linear model with the SPARK total score as dependent variable, testing the two-way interaction between cluster identity and the trait of interest. Contrast analysis was then performed and *p*-values from the models and contrasts were corrected via FDR.

1. **Supplementary** **Figure 1** Multiple correlation matrix


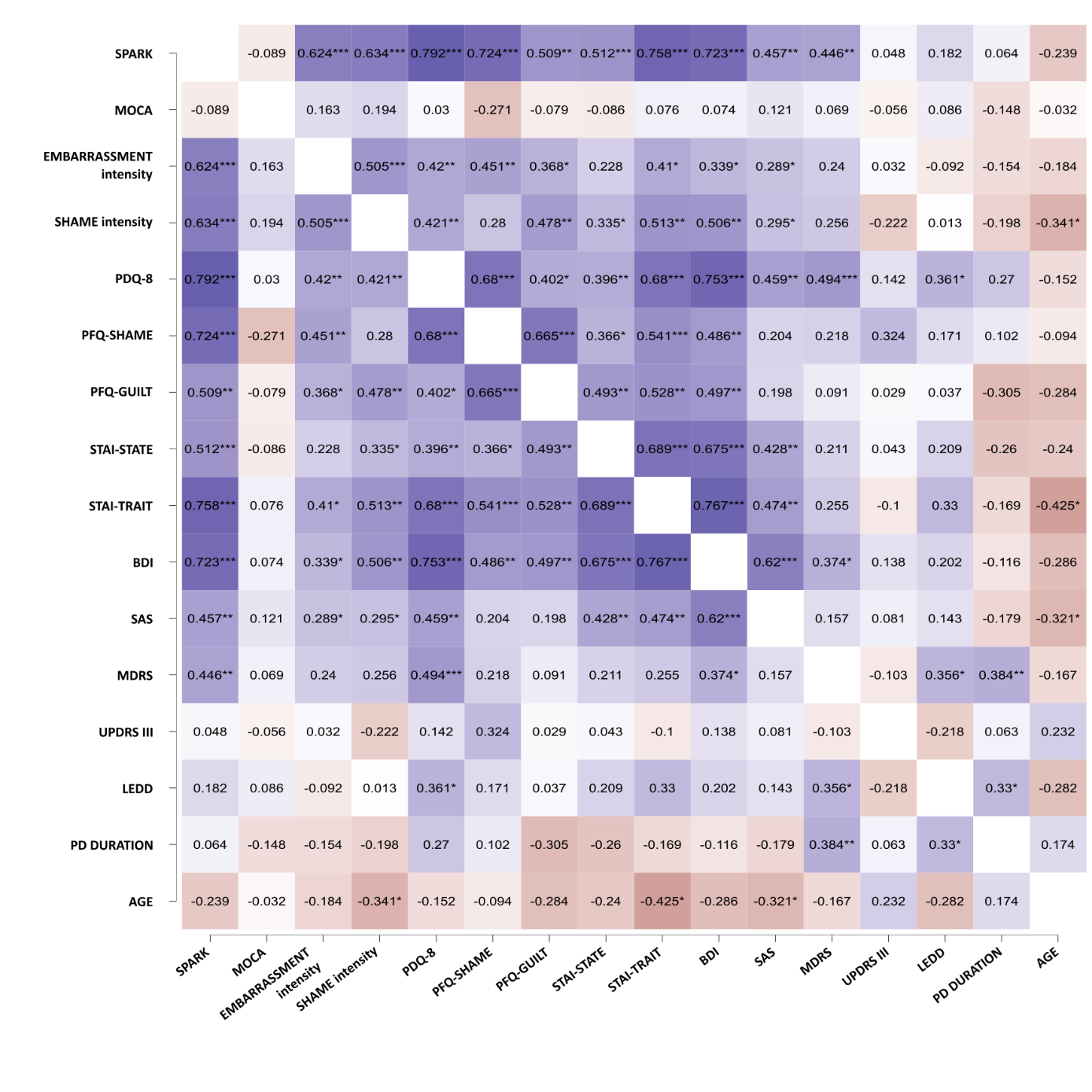


BDI: Beck Depression Inventory; LEDD: levodopa equivalent daily dose; UPDRS III: Unified Parkinson’s Disease Rating Scale Part III; MDRS: Marconi Dyskinesia Rating Scale; MOCA: Montreal cognitive assessment; PDQ-8: Parkinson's Disease Questionnaire; PFQ-2: Personal Feelings Questionnaire with PFQ-Shame scale and PFQ-Guilt scale; SAS: Starkstein Apathy Scale; SPARK: Shame and embarrassment in PARKinson’s disease scale; STAI: State-Trait Anxiety Inventory

1. **Supplementary Figure 2 |** Total SPARK scores across the three patient clusters


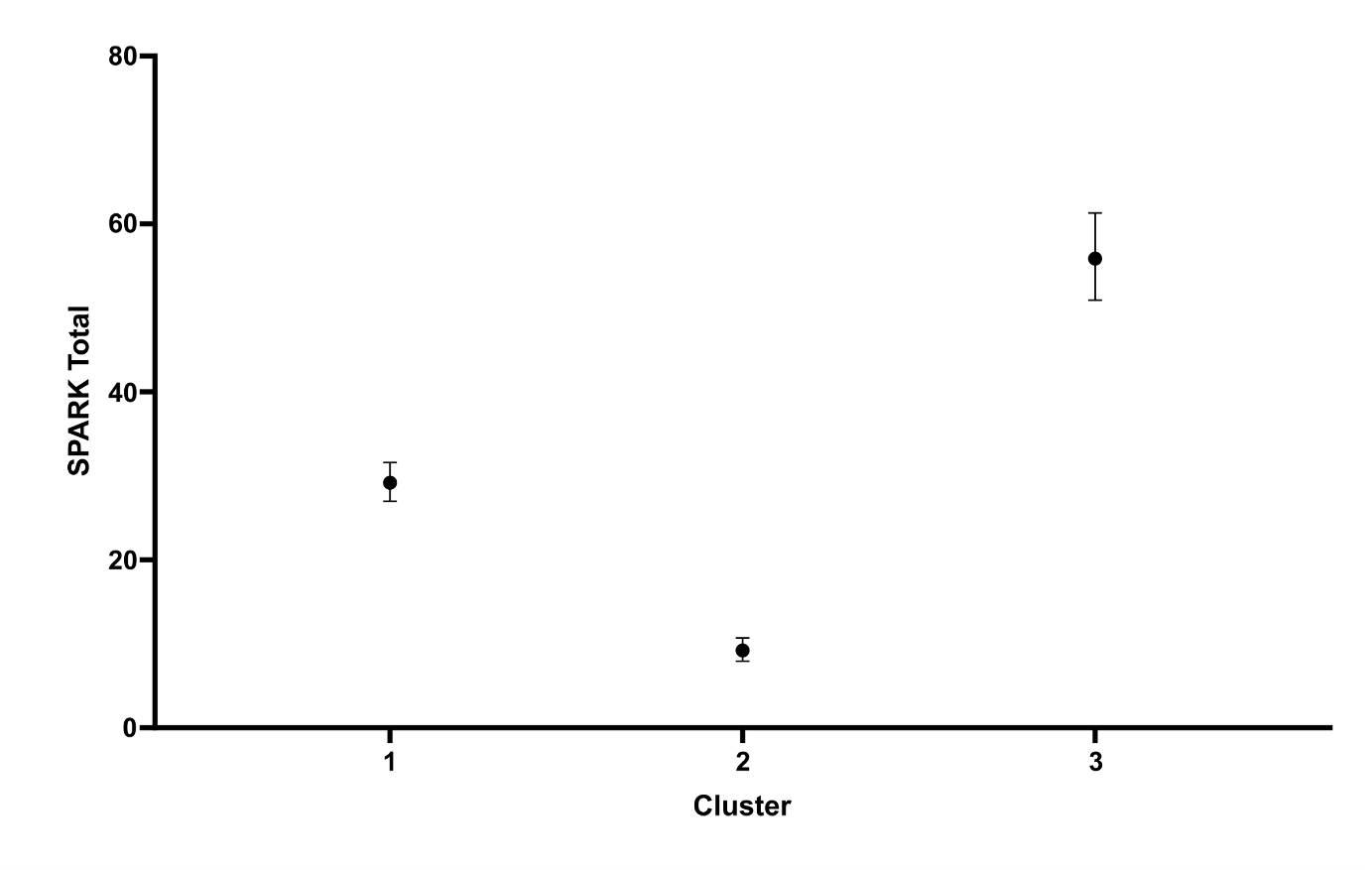


Cluster 1: intermediate total-SPARK score cluster; Cluster 2: lowest total-SPARK score cluster; Cluster 3: highest total-SPARK score cluster

1. **Supplementary Figure 3 |** Distribution of Components 1, 2 and 3 across the three patient clusters


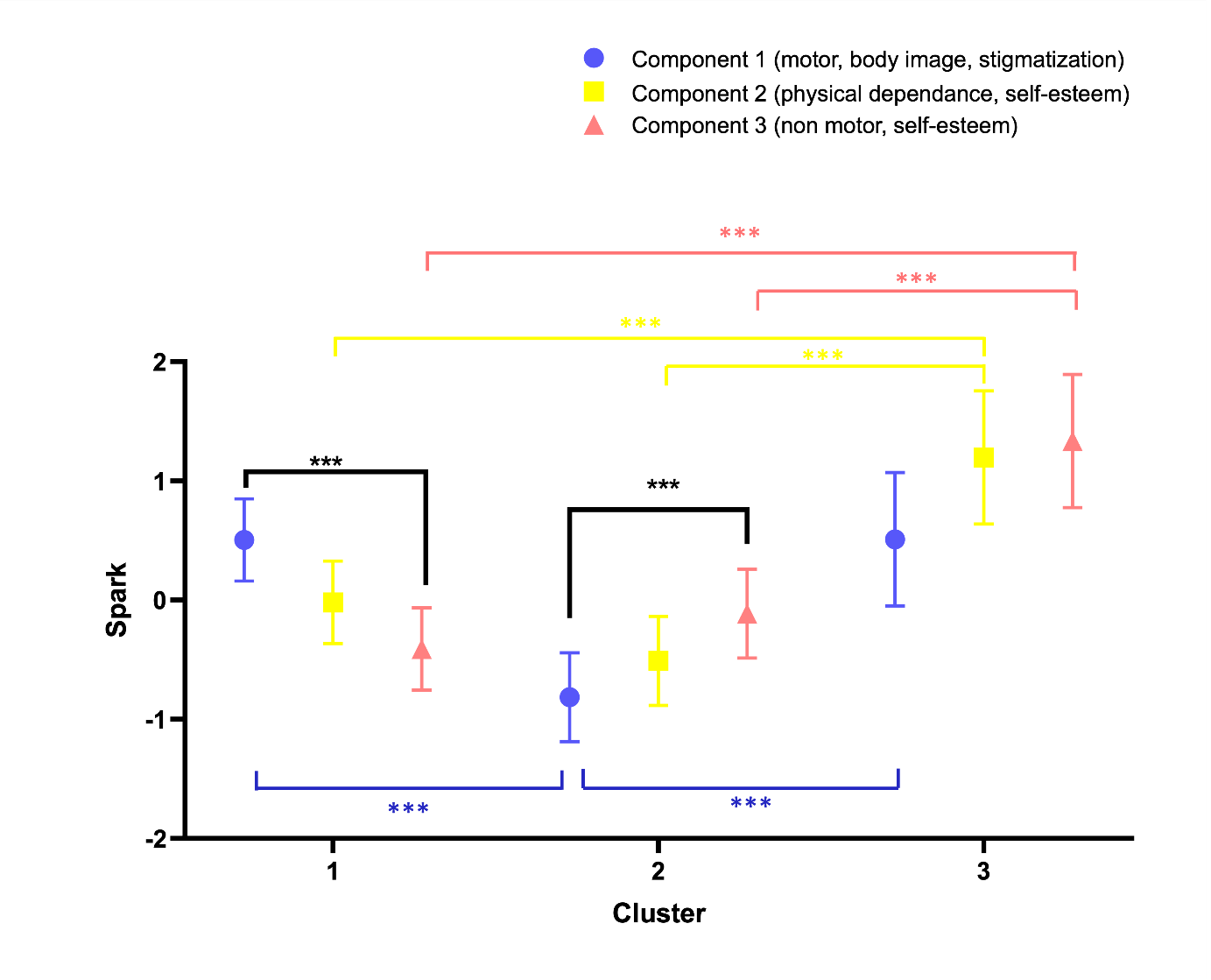


1. **Supplementary Table** Covariation between determinants and patient’s clusters

|  | F | *p* (FDR corrected) |
| --- | --- | --- |
| **Personal determinants** |  |  |
| Age | -1.055 | 0.224 |
| Propensity to feel ashamed (PFQ-2) | **-11.939** | **0.014*** |
| Propensity to feel guilty (PFQ-2) | 3.358 | 0.271 |
| **PD-related determinants** |  |  |
| ***PD characteristics*** |  |  |
| PD duration | -1.120 | 0.445 |
| Hoehn and Yahr stage (/4) | 0.015 | 0.812 |
| ***Clinical symptoms*** |  |  |
| Motor score MDS-UPDRS III (/132) | -0.120 | 0.956 |
| Dyskinesia Rating scale (/28) | **12.266** | **0.005**** |
| Cognitive score MoCA (/30) | 0.011 | 0.812 |
| State-Trait Anxiety Inventory (STAI) |  |  |
| STAI-state (/80) | **-7.674** | **0.001**** |
| STAI-trait (/80) | **-7.674** | **4.73E-06***** |
| Beck Depression Inventory (BDI-II) (/63) | **15.107** | **0.002**** |
| Apathy Starkstein (SAS) (/42) | **-4.217** | **0.020*** |
| Health-related quality of life (PDQ-8) (/8) | **-21.625** | **2.00E-09***** |
| ***Medication*** |  |  |
| Levodopa-equivalent daily dose (mg/d) | -14.702 | 0.956 |

1. **Supplementary Figure 4 |** Correlation between personal and PD-related determinants, and patient clusters


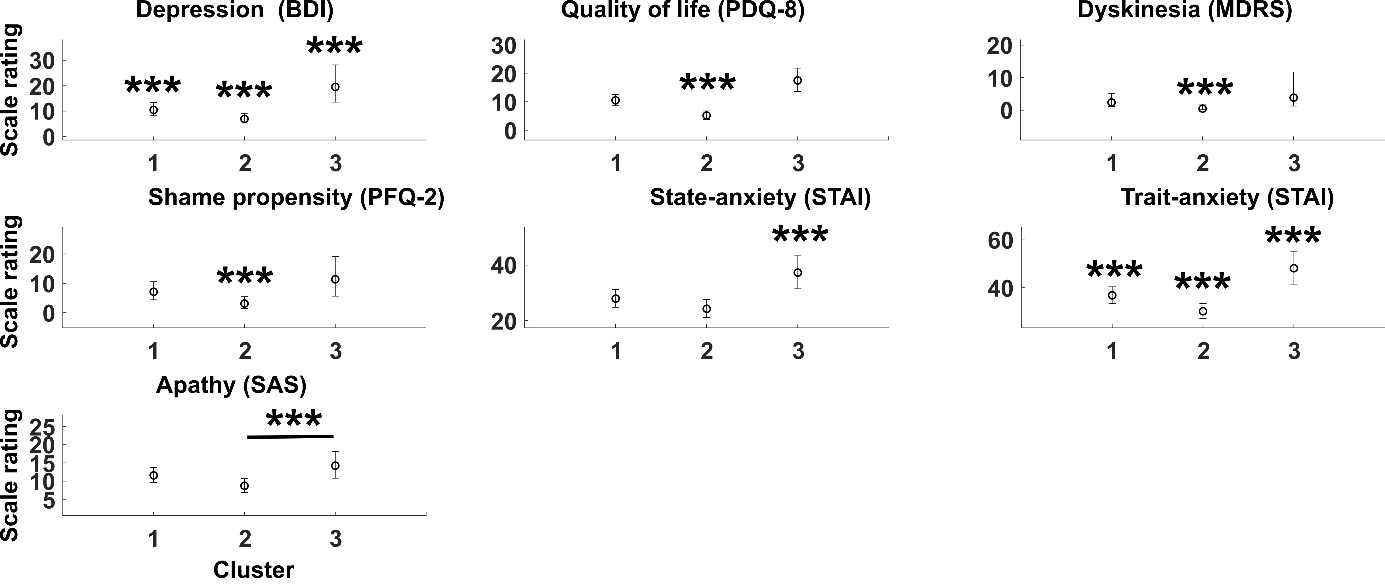


BDI: Beck Depression Inventory; MDRS: Marconi Dyskinesia Rating Scale; PFQ-2: Personal Feelings Questionnaire, PFQ-Shame subscale; SAS: Starkstein Apathy Scale; STAI: State-Trait Anxiety Inventory
